# Post annealing induced manipulation of phase and upconversion luminescence of Cr^3+^ doped NaYF_4_:Yb,Er crystals†

**DOI:** 10.1039/c9ra00115h

**Published:** 2019-03-22

**Authors:** Shivanand H. Nannuri, Suresh D. Kulkarni, Subash C. K., Santhosh Chidangil, Sajan D. George

**Affiliations:** Department of Atomic and Molecular Physics, Manipal Academy of Higher Education Manipal Karnataka India-576104 sajan.george@manipal.edu; Centre for Applied Nanosciences, Manipal Academy of Higher Education Manipal Karnataka India-576104; School of Nanoscience and Technology, National Institute of Technology Calicut India-673601; Centre for Biophotonics, Manipal Academy of Higher Education Manipal Karnataka India-576104

## Abstract

The role of post synthesis annealing at different temperatures (200–600 °C) on the structural as well as luminescence properties of NaY_80%_F_4_:Yb_17%_,Er_3%_ prepared *via* a coprecipitation method was found to change the structure from a cubic to hexagonal phase with a concomitant increase in upconversion luminescence by 12 times for the green region and 17 times for the red region. Addition of the Cr^3+^ ions (5–20 mol%) into the host followed by post annealing at 200–600 °C causes that the samples to exhibit phase dependent and upconversion luminescence behavior that depend upon the doping concentration as well as the annealing temperature. The inductively coupled optical emission spectroscopy reveals that only 1/600 times of the desired volume of the co-dopant goes to the lattice and it can manifest visible spectral changes in the diffuse reflectance spectra of the samples. The samples co-doped with Cr^3+^ ion concentrations of 10–15% and post-annealed at 600 °C were found to have maximum emission with an enhancement factor of 24 for the green region and 33 for the red region. In addition, the laser power dependent studies reveal that even for the power density levels 3.69 W cm^−2^ to 32.14 W cm^−2^, the samples are in the saturation regime and most of the samples investigated here follow a single photon process, and a few samples show a slope value less than 1 for laser power dependent intensity plots. The results show the remarkable promise of controlled tailoring of the properties of upconversion crystals *via* post annealing and co-doping.

## Abstract 2

Co-dopant (Cr^3+^ ion) concentration as well as post annealing found to change the structural as well as luminescence properties of Cr^3+^ ion doped NaY_80%_F_4_:Yb_17%_,Er_3%_ prepared *via* a co-precipitation method.

## Graphical abstract

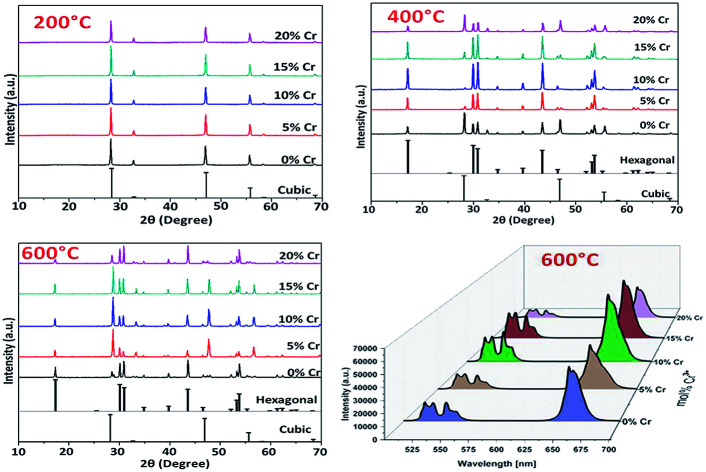

## Introduction

The advancements in nanoscience and nanotechnology have witnessed a concomitant evolution in synthesis of many novel nanoparticles. Among the diverse class of nanoparticles, synthesis and applications of metal nanoparticles and upconversion nanoparticles are attaining much attention recently.^1–10^ Engineering the properties of upconversion nano- and micro particles is gaining much momentum due to their wide spread applications diverse areas including biosensing, optical devices, temperature probes, deep tissue bioimaging, photovoltaics, super-resolution microscopy.^11–17^ This particular unique class of materials can absorb multiple low energy photons *via* real energy levels and emit high energy radiation through the 4f electronic transitions. In stark contrast to the broad absorption and emission bands of the dye molecules, the anti-stoke fluorescence of these particles are relatively sharp. As compared to other popular nano-sized fluoroprobes, quantum dots, the upconversion nanoparticles have multiple absorption and emission centers. In addition, these particles are shown to offer many unique favourable properties such as high photo and thermal stability, little autofluorescence and a non-blinking nature and less toxicity^18,19^ In spite of these advantages, these particles still suffer from low quantum efficiency. Therefore, recent efforts have been focusing on improving the upconversion efficiency *via* co-doping with a sensitizer ion along with an activator ion that have closely matched intermediate excited state. Among the wide range of the dopants and the host matrix reported so far, the sensitizer–activator pair Yb^3+^ and Er^3+^ or Yb^3+^ and Tm^3+^, and β-phase NaYF_4_ is known to be the most promising candidate for luminescence-based applications.^20–22^ The popularity of the particular host matrix stems from the low phonon energy of the host matrix (<400 cm^−1^) and resonance in energy levels for efficient energy transfer to the sensitizer–activator pair.^23^ However, leveraging of the energy transfer process requires proper management of the doping concentration and optimal interaction of sensitizer and activator. Despite a high surface area to volume ratio attained by reducing the size of the particles, a high doping concentration often leads to cross relaxation energy loss and energy migration to the surface quenchers. Many strategies such as coating an inert shell, increasing the excitation power density, choosing a host crystal of large unit cell size, and homogenous distribution of the dopant are adopted to overcome the concentration induced quenching of upconversion materials.^24–26^

On the other hand, various methods such as core–shell structure, varying the crystal phase, incorporation of RE^3+^ ion or non RE^3+^ ion, metal enhanced fluorescence *etc.*, have been utilized to improve the luminescence efficiency of the upconversion materials.^27–29^ It has been proved that changing the crystal field of the host matrix can modulate the electron transition probabilities of RE^3+^ ion and thus control the emission properties of the upconversion materials.^30^ The size and dipole polarizability of lanthanide dopant can be controlled by simultaneous doping with non-RE^3+^ ions of lower or higher ionic radius. Such co-doping of mono, di, and tri valent materials are reported to change the emission properties of upconversion materials.^31–36^ Depending upon the ionic radius difference between the substituted dopant ion and host lattice ion, the co-doping is shown to tailor the phase of the host matrix to cubic/hexagonal phase.^37–40^ Many approaches such as solvothermal method, microwave synthesis route, hydrothermal method, co-precipitation method has shown to be efficient in co-doping of mono, di or tri valent materials into the host matrix.^41–44^ The major challenge with co-doping of the materials is that co-doping suffer from inclusion of very low concentration of co-dopant into the lattice, as evidenced in inductively coupled plasma-optical emission (ICP-OES) studies.^39^ Even though there are some reports that manifest the enhancement in the red and green emission with addition of very low concentration of co-dopant like Cr^3+^ or Mn^2+^, majority of the studies reported till date utilize high concentrations of the co-dopants.^28,39,45,46^ To best of our knowledge, the samples investigated here, the co-dopant Cr^3+^ (for the mol%: 0%, 5%, 10%, 15% and 20%) induced changes in the crystalline size, phase, upconversion luminescence, laser power dependent emission behaviour of NaY_(80−*n*%)_F_4_:Yb_(17%)_,Er_(3%)_Cr_(*n*%)_ (*n* = 0, 5, 10, 15, 20 mol%) has never been reported so far for the samples prepared *via* co-precipitation method. Further, the role of post-annealing at different temperatures (200 °C, 400 °C and 600 °C) on the aforementioned properties are not explored. The ICP-OES studies carried out in the present clearly manifest a linear increase incorporation of the Cr^3+^ ions into the host lattice but with a very low concentration compared to the theoretically estimated inclusion of Cr^3+^ ions. Nevertheless, the diffuse reflectance studies exhibit a corresponding change for even low concentration of co-dopant whereas the XRD studies elucidate that annealing temperature as well as co-dopant concentration have pronounced effect on the diffraction peaks of the samples. The X-ray photoelectron spectroscopy (XPS) studies carried out to confirm the oxidation state of the Cr^3+^ ions. The luminescence studies carried out on the samples under 980 nm excitation elucidate that co-dopant concentration and post annealing temperature play a critical role in obtaining the enhancement luminescence signal of the sample. The laser power dependent study unravels that, contrary to many reported studies, most samples investigated here follows single photon process while some of them exhibit a slope value less than 1 for the double logarithmic plot of green and red intensity against laser power density.

## Experimental

### Materials and methods

Rare earth nitrates Y(NO_3_)_3_·*x*H_2_O (99.9%), Yb(NO_3_)_3_·*x*H_2_O (99.9%), Er(NO_3_)_3_·5H_2_O (99.9%), were purchased from Alfa Aesar (http://www.alfa.com). EDTA are purchased from Merck India. NaF and CrCl_3_·6H_2_O (98%) are procured from Sigma Aldrich and HiMedia Laboratories Pvt. Ltd, India, respectively. All other reagents are of analytical grade and are used directly without further purification.

### Synthesis of Cr^3+^ doped NaYF_4_:Yb,Er crystals by coprecipitation method

NaY_(80−*n*%)_F_4_:Yb_(17%)_,Er_(3%)_Cr_(*n*%)_ (*n* = 0, 5, 10, 15, 20 mol%) crystals were prepared *via* co-precipitation method using EDTA as a capping ligand and surface modifier. At first, for the synthesis of UCNPs NaY_(80%)_F_4_:Yb_(17%)_,Er_(3%)_, solutions were prepared by dissolving Y(NO_3_)_3_ (0.2 M) in 32 ml of DI water, Yb(NO_3_)_3_·*x*H_2_O (0.2 M) in 6.8 ml DI water, Er(NO_3_)_3_·5H_2_O (0.2 M) in 1.2 ml DI water and (0.2 M) of EDTA in 40 ml of DI water. All these solution are mixed well together and named as solution A. Another solution was prepared by dissolving the 4.2 g of NaF in 120 ml DI water. The dissolved NaF solution was added dropwise to the lanthanide mixture solution (solution A) and stirred continuously for 1 hour. The final product was then separated out by centrifugation at 8000 rpm and washed several times with water and finally with ethanol. The sample was dried using lyophilizer and used for further characterizations. For the synthesis of Cr^3+^ ion doped UCNPs NaY_(80−*n*%)_F_4_:Yb_(17%)_,Er_(3%)_Cr_(*n*%)_ (*n* = 0, 5, 10, 15, 20 mol%) CrCl_3_·6H_2_O was used in different mol concentrations, as mentioned above. As synthesized particles did not show any appreciable up-conversion luminescence. However, after annealing process (200°C-600 °C) for 5 h, bright up-conversion fluorescence was observed under the 980 nm infrared excitation.

### Characterization

The amount of Cr^3+^ ions in the samples were investigated using the ICP-OES technique operating in the acid digestion mode calibrated in the 0.25 to 2 ppm under an environmental conditions of 50% humidity and at 20 °C. The diffuse reflectance studies are carried out using a commercially available UV-VIS-NIR spectrophotometer (Perkin Elmer Lambda 950). The samples were annealed by placing the samples in a muffle furnace. The crystallographic phase of the samples under investigation were characterized by the powder XRD on a X-ray diffractometer (Rigaku Ultima IV) with CuKα radiation operating at *λ* = 1.5046 Å at 2 degree per min in the angle 10–70°. The XPS studies the oxidation state of the Cr was investigated using XPS instrument (PHI Versaprobe III). The field enhanced scanning electron microscope images were taken using Zeiss FESEM instrument (Hitachi SU 6600) at 5 kV with 15k magnification. The photoluminescence spectra were collected using a home-built experimental setup with 980 nm laser as excitation source. The excitation laser was coupled to the powdered sample through a 200 μm central fiber and the surrounding six 200 μm fibers are used to collect the sample (ESI Fig. S1†). The distance between the fiber probe and the sample is kept at ∼3.25 mm.

## Results and discussions

**Fig. 1:**
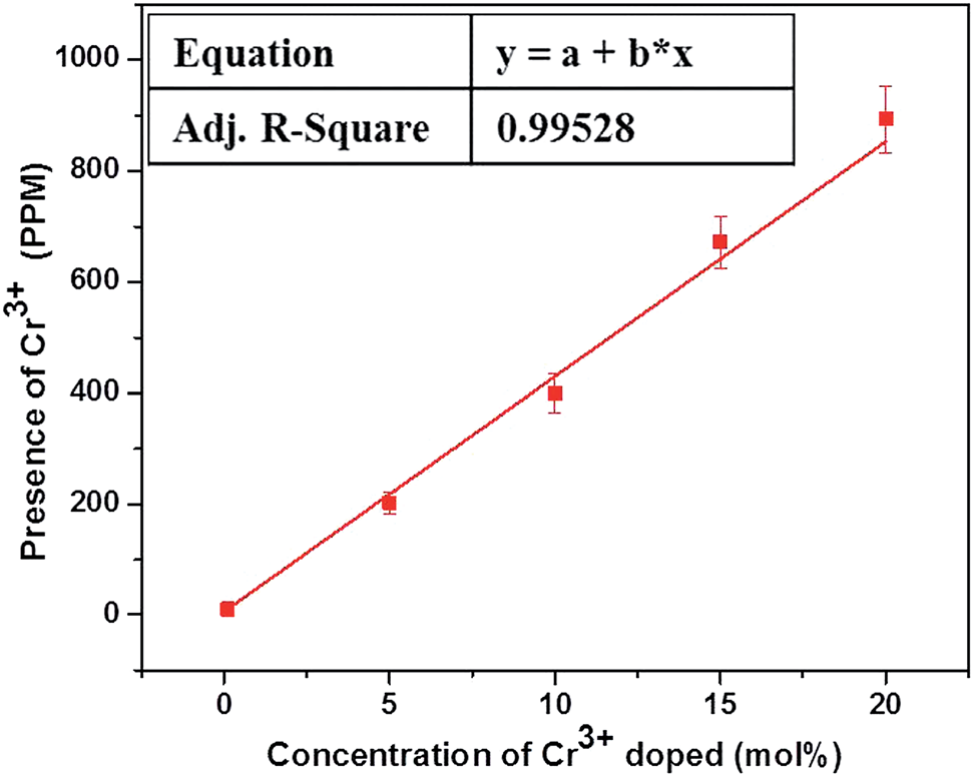
ICP-OES studies of Cr^3+^ codoped NaYF_4_:Yb^3+^/Er^3+^ crystals annealed at 600 °C.

The actual concentration of Cr^3+^ ions for the samples annealed at 600 °C is measured using the ICP-OES studies and the results are shown in Fig. 1. It is worthwhile to note here that, irrespective of the high concentration used here, the actual concentration of the Cr^3+^ ions in the lattice are much smaller than the desired volume. Nevertheless, the ICP-OES results confirm the successful co-doping of Cr^3+^ ions into the host lattice. It is pertinent note here that, despite the linear increase in the successfully co-doped Cr^3+^ ions with the original volume taken, only ∼1/600 times Cr^3+^ ions really gone into the lattice and thus demand careful evaluation of actually co-doped concentration in the lattice when studies are carried out with very low concentration of the co-dopant, as reported in many previous studies.

**Fig. 2:**
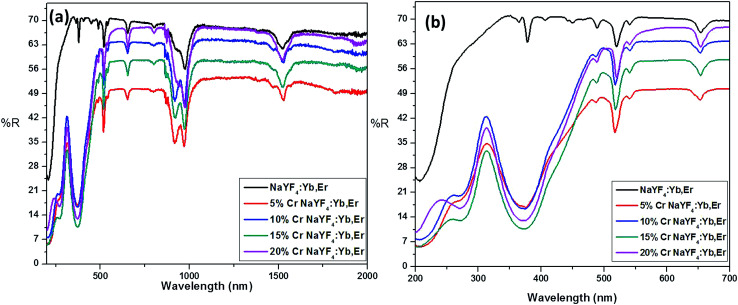
Diffuse reflectance spectra of Cr^3+^ codoped NaYF_4_:Yb^3+^/Er^3+^ crystals annealed at 600 °C.

The incorporation of Cr^3+^ ions into the host lattice is further confirmed by the diffuse reflectance studies, as shown in Fig. 2. The co-doped samples clearly manifest a drastic change in the reflectance spectra, especially for the enlarged region shown in Fig. 2b. The two noticeable bands at 271 nm and 376 nm can be assigned to the ^4^A_2g_(F) → ^4^T_1g_(P), ^4^A_2g_(F) → ^4^T_1g_(F) allowed transitions of Cr^3+^ ions.^47^ The FESEM studies carried out for the samples annealed at 600 °C with different concentrations of Cr^3+^ ion (ESI S2†).

**Fig. 3:**
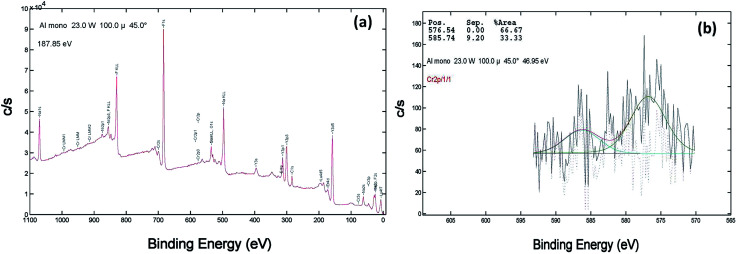
XPS data of 10 mol% Cr^3+^ codoped NaYF_4_:Yb^3+^/Er^3+^ crystals annealed at 200 °C (a) survey data (b) the deconvolution of the Cr2p spectrum.

**Fig. 4:**
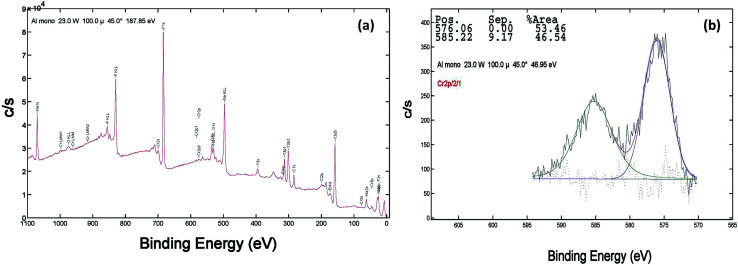
XPS data of 10 mol% Cr^3+^ codoped NaYF_4_:Yb^3+^/Er^3+^ crystals annealed at 400 °C (a) survey data (b) the deconvolution of the Cr2p spectrum.

**Fig. 5:**
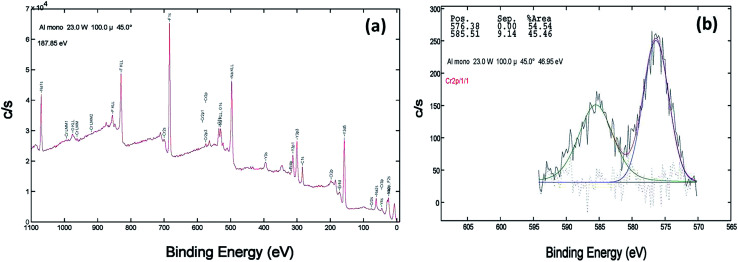
XPS data of 10 mol% Cr^3+^ codoped NaYF_4_:Yb^3+^/Er^3+^ crystals annealed at 600 °C (a) survey data (b) the deconvolution of the Cr2p spectrum.

XPS of the Cr doped NaYF_4_:Yb,Er crystals annealed at 200–600 °C samples was studied using AlKα radiation and the broad spectral feature (survey spectra) of a typical sample is shown in Fig. 3a, 4a and 5a. The spectra show the presence of several elements (Na, Y, Yb, Er, F, Cr). The de-convoluted high resolution core level spectra of Cr2p states are shown in Fig. 3b, 4b and 5b respectively. The Cr2p spectra when de-convoluted (Fig. 3, 4 and 5(b)) produce two peaks respectively at ∼576 eV and ∼585 eV corresponds to Cr^3+^.^48,49^

**Fig. 6:**
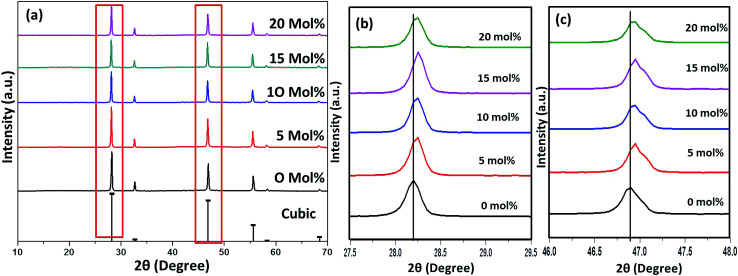
(a) XRD spectra of Cr^3+^ codoped NaYF_4_:Yb^3+^/Er^3+^ crystals annealed at 200 °C (b) enlarged XRD patterns of the diffraction peaks (111). (c) Enlarged XRD patterns of the diffraction peaks (2 2 0).

**Fig. 7:**
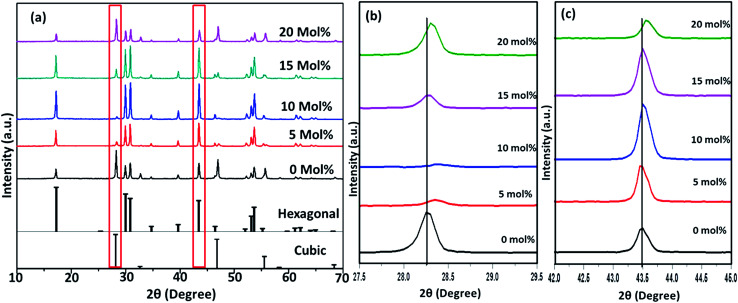
(a) XRD spectra of Cr^3+^ codoped NaYF_4_:Yb^3+^/Er^3+^ crystals annealed at 400 °C (b) enlarged XRD patterns of the diffraction peaks (111). (c) Enlarged XRD patterns of the diffraction peaks (2 0 1).

**Fig. 8:**
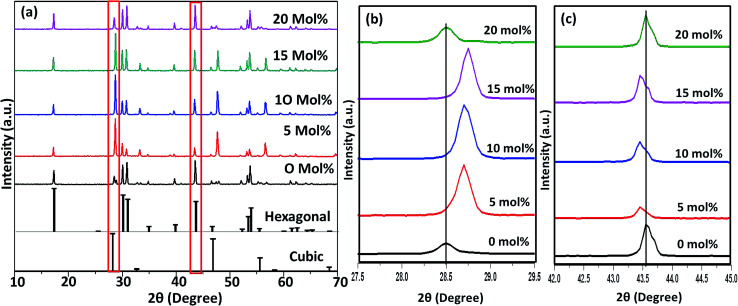
(a) XRD spectra of Cr^3+^ codoped NaYF_4_:Yb^3+^/Er^3+^ crystals annealed at 600 °C (b) enlarged XRD patterns of the diffraction peaks (111). (c) Enlarged XRD patterns of the diffraction peaks (2 0 1).

Fig. 6–8(a) shows the X-ray diffraction peaks of the as prepared NaYF_4_:Yb_(17%)_,Er_(3%)_ samples doped with 0–20 mol% of Cr^3+^ ions annealed at 200 °C, 400 °C and 600 °C, respectively. It is clear from Fig. 6(a) that, for the samples annealed at 200 °C, with and without Cr^3+^ ion doping, the samples are in the cubic phase of NaYF_4_:Yb,Er (JCPDS01-77-2042). However, with the incorporation of the Cr^3+^ ions, the diffraction peak corresponding to (111) and (220) shifted to higher Bragg angles, as shown in Fig. 6(b) and (c) respectively. The substitution of Cr^3+^ ion of lower ionic radius (62 pm) in place of the Y^3+^ (90 pm) ions in the host lattice lead to the contraction of the host lattice and result in decreased unit cell volume and decrease in inter-planar distance, resulting in the experimentally observed shift in the diffraction peak shift. When the sample was annealed at 400 °C, the NaYF_4_:Yb_(17%)_,Er_(3%)_ exhibit a mixed phase of cubic and hexagonal phase (JCPDS00-28-1192). The phase transition from cubic to hexagonal and the reversal to cubic phase with increasing temperature has already been reported for similar samples.^43^ In our case of sample annealed at 400 °C with 10% doping of Cr^3+^ ions, the sample completely changes to the hexagonal phase and further addition of the co-dopant (15%) results in a mixed phase of cubic and hexagonal phase with dominating hexagonal phase. However, it is observed that co-doping of 20% Cr^3+^ ions exhibits a mixed phase with cubic phase dominating, indicating the critical role of co-dopant concentration on the phase of the sample annealed at a given temperature. As shown in Fig. 7(b) and (c), aside the intensity change in the cubic and hexagonal phase peak, the diffraction peaks corresponding to (111) and (201) crystal face exhibits clear shift to the right side for the sample co-doped with 20% of Cr^3+^ ions and implies that the substitutional doping happening in this case. The annealing of sample to higher temperature 600 °C results in a mixed phase with dominating hexagonal phase of NaYF_4_:Yb_(17%)_,Er_(3%)_. However, tri-doped samples annealed at this temperature shows mixed phase with predominant cubic phase, except for 20% of Cr^3+^ ions. For this higher concentration annealed at 600 °C, the sample remains in a mixed phase but predominantly hexagonal phase. It is thus evident that the co-dopant concentration also plays a critical role in determining the phase of the prepared crystal. Interestingly, as shown in Fig. 8(b), the co-doped samples exhibiting cubic dominating phase shows a shift towards the right-side for the diffraction peak correspond to (111) crystal phase but in the case of co-existing hexagonal phase shows a shift towards the left-side for the diffraction peak correspond to (201) crystal phase as shown in Fig. 8(c). It may be due to the fact that at these concentrations; the co-dopant goes to the interstitial lattice positions and results in the expansion of the lattice and unit cell volume.^39^ It is imperative to mention here that both substitution and interstitial doping can tailor the crystal electric field around the luminescence element Er^3+^ ion can give rise to the transition between the energy levels which is otherwise forbidden. The summarized pseudo phase diagram obtained from the XRD data is shown in Fig. S3.†

**Fig. 9:**
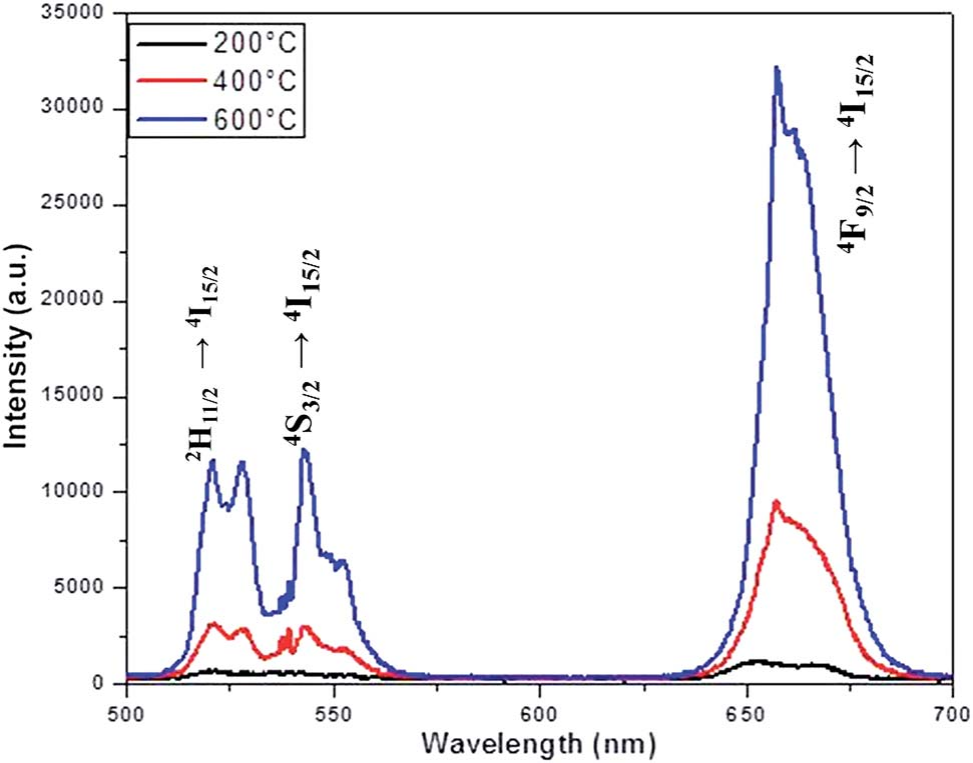
Emission spectra of NaYF_4_:Yb^3+^/Er^3+^ crystals annealed at different temperature.

**Fig. 10:**
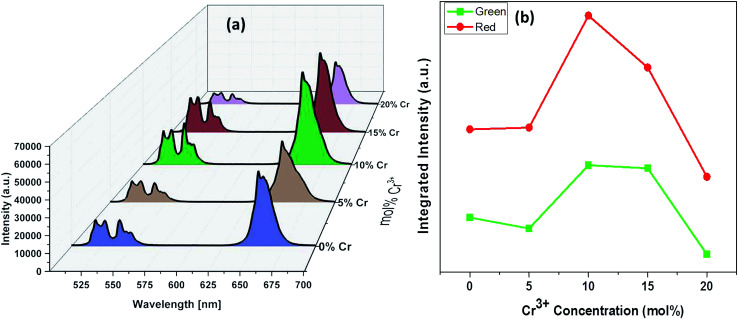
(a) Emission spectra of *a*YF_4_:Yb^3+^/Er^3+^ crystals annealed at 600 °C with respect to Cr^3+^ concentrations. (b) Plots of integrated intensity *versus* the concentrations of Cr^3+^ ions.

The luminescence spectra measured for the NaYF_4_:Yb_(17%)_,Er_(3%)_ clearly manifest that the crystals synthesized here exhibit characteristic emission bands in green and red region, which we label them as G1 (centred around 525 nm corresponding to ^2^H_11/2_ to ^2^I_15/2_) G2 (centred around 540 nm corresponding to ^4^S_3/2_ to ^2^I_15/2_) and R (centred around 655 nm corresponding to ^4^F_9/2_ to ^2^I_15/2_), as shown in Fig. 9. It is worthwhile to mention here that the as synthesized samples show little luminescence. However, the samples exhibit luminescence that increases with the annealing temperature. The emission of G1, G2, G (combined G1 and G2) is increased by 12 times by changing the annealing temperature from 200 °C to 600 °C whereas the enhancement is 17 times for R. The enhancement factor for the samples co-doped with Cr^3+^ ions obtained from the supplementary Fig. S4† are given in Table 1. It is evident from the Table 1 that inclusion of Cr^3+^ ions up to 10–15% have positive influence on the overall luminescence in the green as well as red emission region of the crystal and further addition have negative influence on the enhancement of the luminescence signal strength. This implies that, irrespective of the dominant hexagonal phase of 20% Cr^3+^ ions co-doped samples, the enhancement is less as compared to the dominant cubic phase observed in the case of 10 and 15% co-doped samples. It is well known that the emission intensity of lanthanide doped upconversion materials are affected by many factors including lanthanide doping concentration, shape, size and host structure of the particle. The Cr^3+^ ion doping provides new energy levels for the energy transition which can alter the upconversion process by promoting the red and/or green emission. The intra-4f transition probability of lanthanide activator Er^3+^ ion and the surface quenching are determining factor in the luminescence emission. It is expected that annealing at high temperature lead to not only better crystallinity but also reduce surface quenching and thus exhibit experimentally observed enhancement in luminescence. In addition, we observe that the enhancement factor relies on the co-dopant concentration also. The luminescence quenching at high doping concentration of upconversion materials are normally attributed to increased occurrence of energy transfer between the dopants. The high doping can lead to the energy migration of excited levels within the sensitizer–sensitizer network to the quenchers in the surface. Moreover, the inter-dopant cross relaxation between the activators leads to decrease in luminescence. The same valency of the Cr^3+^ ion and Y^3+^ ion avoid the charge balance issue and the doping site issue. However, for a given sensitizer and activator concentration as in our case, the addition of co-dopant alters the host lattice unit cell parameters and causes the distortion of the lattice which affects the spatial distribution of the lanthanide ions, inducing a concentration quenching and reduction in the emission intensity, as reported in many previous studies. The decrease in luminescence is predominant in green region as compared to the red region may arise from the fact that, unlike green emission, the red emission happens *via* several possible excitation mechanisms.^50,51^ Recent studies claim that non-multiphoton relaxation and cross relaxation between dopant and Er^3+^ plays an important role in red emission.^52,53^ It is evident in Fig. 10 that the co-doped samples with various mol percentages of Cr^3+^ions annealed at 600 °C have substantial influence of the luminescence behaviour. The upconversion emission intensities in the green and red regions are slightly decreased with 5 mol% of Cr^3+^ ions but shows a substantial increase when 10 mol% of Cr^3+^ ions are added.

**Table 1:** The enhancement factor for the samples co-doped with Cr^3+^ ions

Cr^3+^ ion concentration (in mol%)	G_600_/G_200_	R_600/_R_200_
0	12	17
5	18	24
10	24	32
15	24	33
20	12	26

To further understand the UC process is saturated or unsaturated, the laser power density dependent emission behaviour of green and red region of the samples investigated here are measured and it is expected that the steady state population density of a state and hence the upconversion luminescence intensity (*I*) should follow *I* ∝ *P*^*n*^, where *P* is the laser power density and *n* is the number of laser photons absorbed per upconverted photon emitted. Herein, we measured the laser power dependent emission of the samples with various concentrations of co-dopant and post-annealed at different temperature. The integrated green and red upconversion luminescence intensity for the power densities from 3.69 W cm^−2^ to 32.14 W cm^−2^ is measured for all the samples under investigation and shown in supplementary Fig. S5–S9.† From the double logarithmic plot between the integrated intensity in the green and red region against the power density, the slope *n* is estimated and the results for all the samples investigated here are shown in Table 2. It is interesting to mention here that, contrary to many two or three photon process reported in literature, the slope of green as well as red region is most of the samples are nearly 1 and a few samples show slope less than 1. Thus the samples can be considered to be in the saturation effect regime. In situations wherein the upconversion emission is excited by the sequential absorption and energy transfer of *n* photons, its dependence on absorbed pump power decreases from *P*^*n*^ down to *P*^1^ with increasing excitation power, as observed here. This linear dependency applies normally for highest energy electronic states that is excited *via* upconversion process. On the other hand, the lower lying levels (excited by *m* photons, where *m* < *n*), is expected to exhibit power dependence that decreases from *P*^*m*^ down to *P*^(*m*/*n*)^, that exhibit slope less than 1.^54^ In the latter case, emission from the intermediate states occur *via* energy transfer upconversion (ETU) and pump excited state absorption (ESA) process. One of the reasons attributed to the decrease in slope is the rise in sample temperature due to increase in non-radiative transitions with increase in laser power.^55^ The phonon assisted energy transfer and multi-phonon relaxation contribute to the non-radiative part of the rare earth elements and it is governed by the energy gap law or phonon number law. With the increase in pump power density, the temperature at the irradiation region increases and causes the decrease in probability of ESA. The laser induced heating of the lattice reported to exhibit a quenching in emission intensity for green and red region for the power density above threshold density.^56^ However, such a reversal in luminescence intensity is not observed in our case. In our case, under 980 nm excitation, the Yb^3+^ ions absorb nearly all the exciting photons (sensitizer) and transfer them to Er^3+^ ions (activator) to manifest the observed emission bands. In the unsaturated case, the energy transfer (ET1) between the excited state of Yb^3+^ populate the ^4^I_11/2_ state of Er^3+^ which further excited to ^4^F_7/2_ state of the Er^3+^*via* energy transfer process (ET2). The nonradiative multiphonon relaxation takes the particle from ^4^F_7/2_ to ^2^H_11/2_/^4^S_3/2_ of Er^3+^ ions and the radiative transition from these two states to the ground ^2^I_15/2_ state of Er^3+^ results in the experimentally observed two emission bands in the green region. This process is a two photon process and expected to give a slope value ∼2. However, the saturation of the excited level lead to the reduction in the slope value. In addition, in general, the upconversion process include the ground state absorption of Yb^3+^ and Er^3+^ ions, energy transfer between Yb^3+^ and Er^3+^ ions, the excited state absorption (ESA) of Er^3+^ ions, and the cross relaxation (CR) between two nearby Er^3+^ ions. This can also account for the observed reduction in the slope value. The co-dopant as well as the doping as well as annealing temperature can alter the host crystal symmetry and thus alter the net excited state dynamics [nonlinear spectral and lifetime management in upconversion crystals by controlling energy distribution] and lead to slope values less than 1.

**Table 2:** Slope values from the double logarithmic plot between the integrated intensity in the green and red region against the power density

Cr^3+^ ion concentration (in mol%)	Samples annealed at 200 °C		Samples annealed at 400 °C		Samples annealed at 600 °C	
	Green (G)	Red (R)	Green (G)	Red (R)	Green (G)	Red (R)
0	1.205	0.9642	1.086	0.9484	1.0928	1.062
5	1.1479	0.799	0.8625	1.021	1.172	1.038
10	0.8742	0.4187	0.9102	1.085	1.142	1.1162
15	1.103	0.6541	0.9124	1.0171	0.9886	0.9157
20	1.2069	0.65272	1.0392	0.966	0.9404	0.7294

## Conclusions

The post annealing temperature and the co-dopant concentration found to play critical role on the phase of the NaY_80%_F_4_:Yb_17%_,Er_3%_ crystals prepared *via* co-precipitation method. Though increasing in annealing temperature from 200 °C to 600 °C changes the phase of NaY_80%_F_4_:Yb_17%_,Er_3%_ from cubic (α) to hexagonal (β) dominating mixed phase, the co-doped samples shows a different behaviour wherein the co-dopant concentration as well as annealing temperature determine the phase of the crystals. The inductively coupled plasma-optical emission spectroscopy unambiguously confirms the presence of co-dopant in the lattice but with 1/600 times less the desired volume. Nevertheless, the diffuse reflectance studies exhibit a concomitant change in the spectra and ensure the presence of co-dopant in the lattice. Despite the dominating cubic (α), the samples annealed at annealed at 600 °C and a co-dopant concentration of 10–15% of Cr^3+^ ions found to be most efficient in enhancing the upconversion luminescence of NaY_80%_F_4_:Yb_17%_,Er_3%_ crystals (24 times for green region and 33 times for red region). The laser power dependent studies show that most of the samples investigated here follows a single photon process instead of expected multi-photon upconversion process and depending upon the co-dopant concentration and annealing temperature, many samples have a slope value less than 1 for double logarithmic plot for upconversion luminescence and power density.

## Conflicts of interest

There are no conflicts to declare.

## Supplementary Material

RA-009-C9RA00115H-s001
