# Supplementary material for: Post annealing induced manipulation of phase and upconversion luminescence of Cr3+ doped NaYF4:Yb,Er crystals
Source: RSC Adv. 2019 Mar 22;9(17):9364–72. doi: 10.1039/c9ra00115h (PMC9062064; doi:10.1039/c9ra00115h)
Supplement: RA-009-C9RA00115H-s001 [file RA-009-C9RA00115H-s001.pdf]

Electronic Supplementary Material (ESI) for RSC Advances.
This journal is © The Royal Society of Chemistry 2019

Supplementary Material (ESI)

Post annealing induced manipulation of phase and upconversion luminescence of Cr^{3+} doped $NaYF_4$:Yb,Er crystals†

Shivanand H. Nannuri,[a] Suresh D. Kulkarni,[a,b] Subash C.K.,[c] Santhosh Chidangil,[a,d] and Sajan D. George,[a,b]

[a]Department of Atomic and Molecular Physics, Manipal Academy of Higher Education, Manipal, Karnataka, India - 576104
[b]Centre for Applied Nanosciences, , Manipal Academy of Higher Education, Manipal, Karnataka, India – 576104
[c]School of Nanoscience and Technology, National Institute of Technology, Calicut, India – 673 601
[d]Centre for Biophotonics, Manipal Academy of Higher Education, Manipal, Karnataka, India - 576104

Contents

- Experimental setup for emission collection
- FESEM Images
- Summarized pseudo phase diagram (T vs mol% Cr)
- Emission spectra of Cr^{3+} doped $NaYF_4$:Yb, Er annealed at different temperatures
- Laser power density dependent emission spectra of all samples.

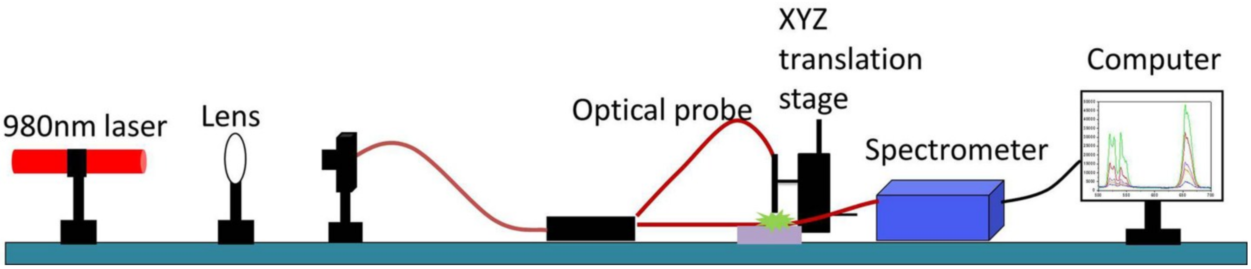

Fig.S1. Fiber coupled excitation-collection laser (980 nm) based experimental setup.

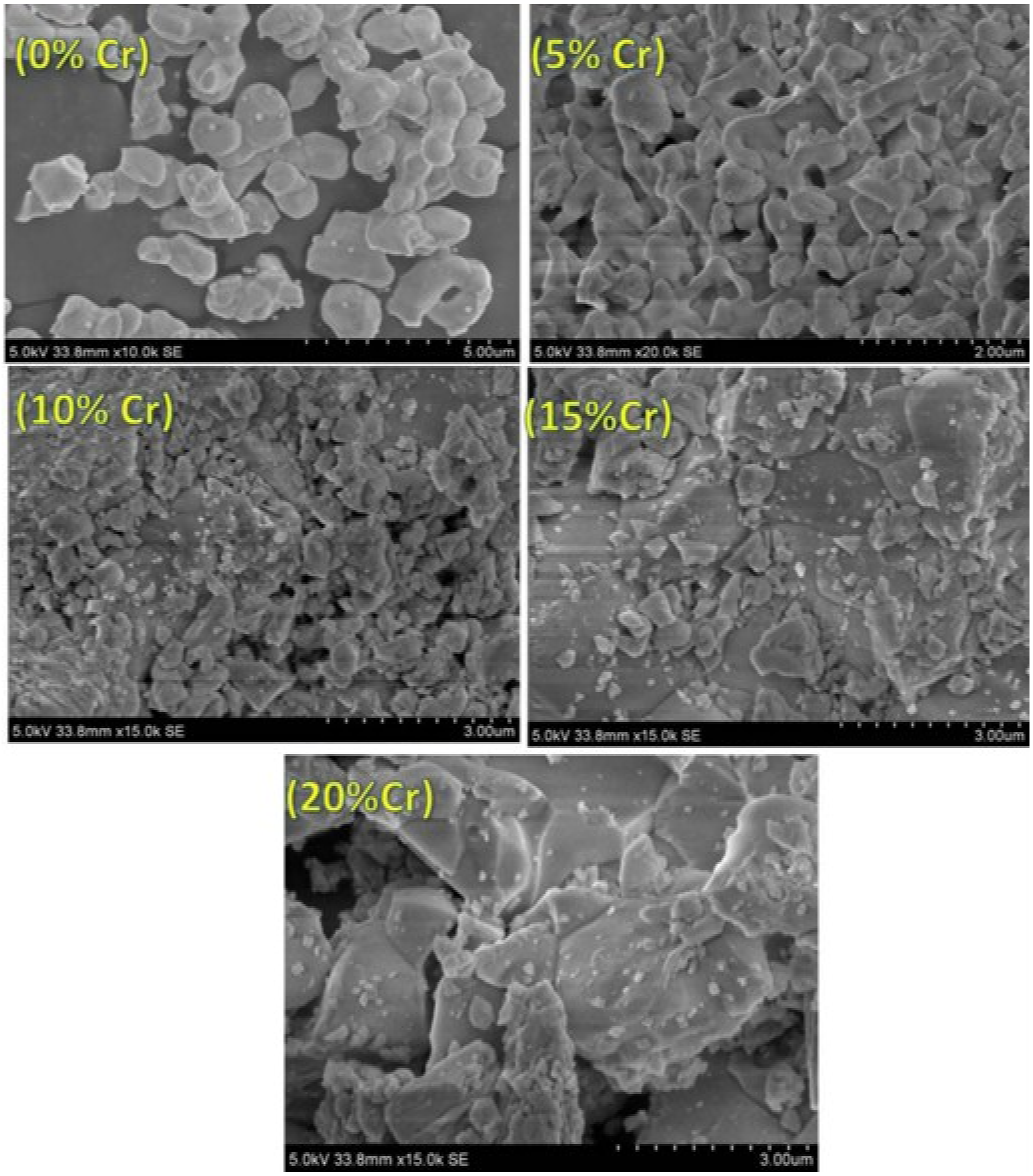

Fig.S2. FESEM images of the NaYF:Yb^{3+}/Er^{3+} crystals codoped with different amount of Cr^{3+} ions annealed at 600 °C : (a) 0 mol%, (b) 5 mol%, (c)10 mol%, (d)15 mol%, (e) 20 mol%.

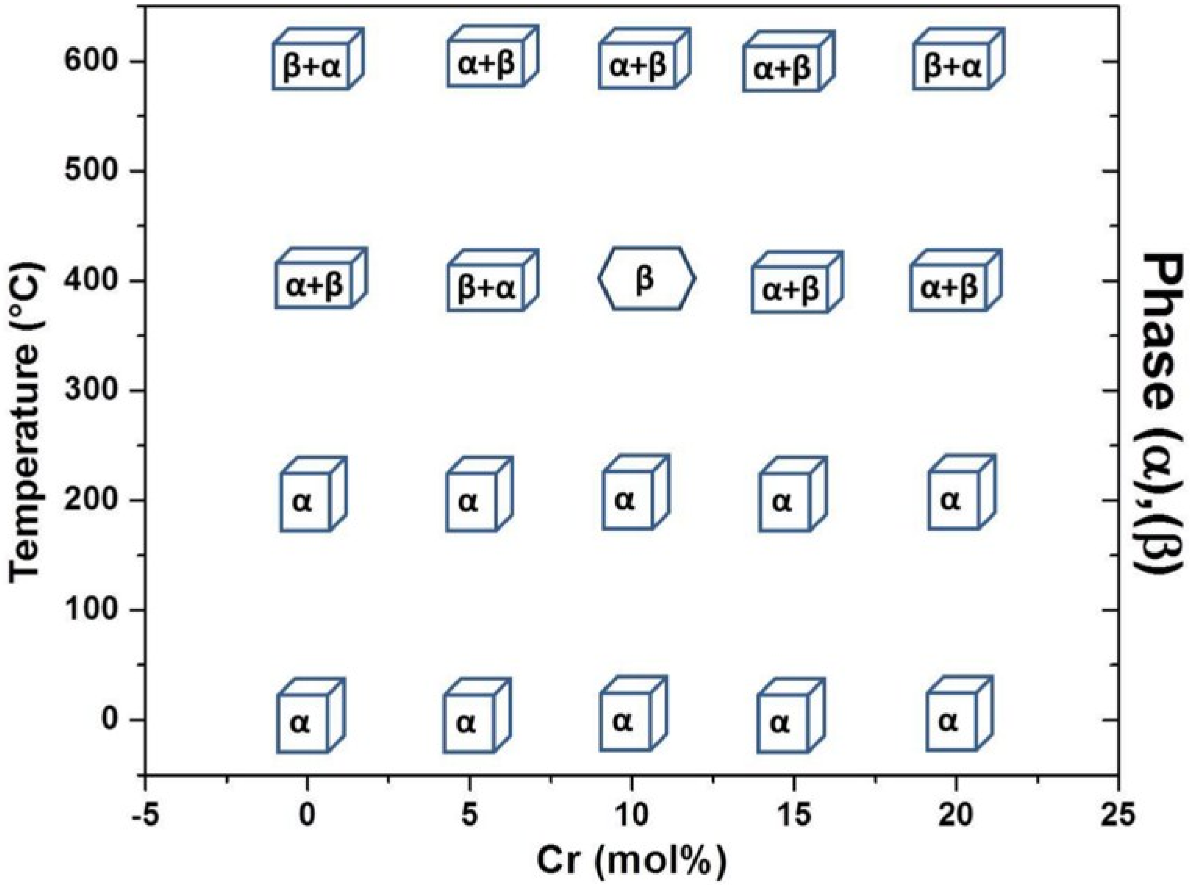

Fig.S3. Summarized pseudo phase diagram (T vs mol% Cr)

Fig.S3 Show the summarized pseudo phase diagram obtained from the XRD data. Here α refers to the pure cubic phase, β refers to pure hexagonal phase, (α+ β) and (β+α) refers to cubic phase dominant mixed phase and hexagonal phase dominant mixed phase respectively.

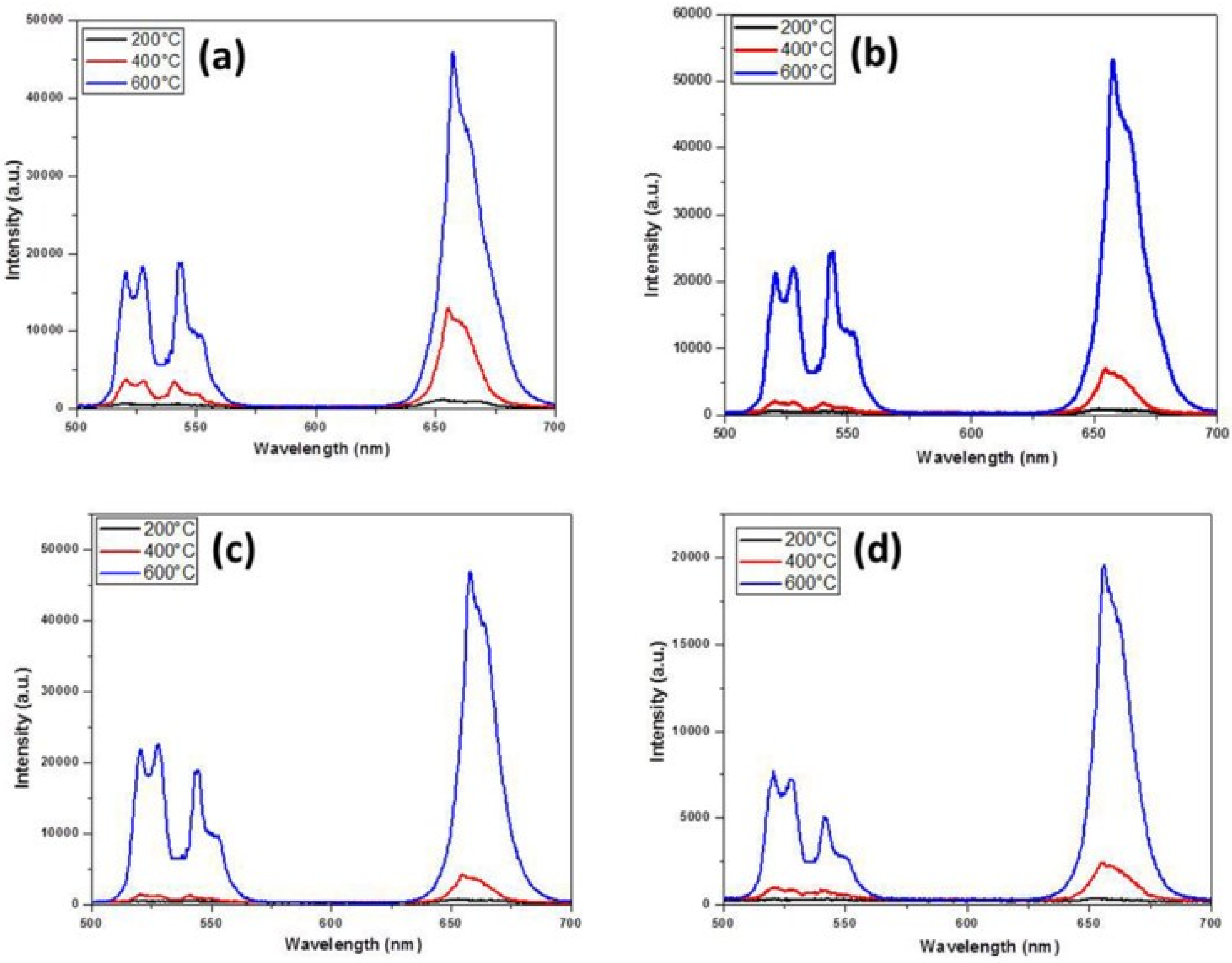

Fig.S4. Emission spectra of the $NaYF_4$:Yb^{3+}/Er^{3+} crystals codoped with different amount of Cr^{3+} ions annealed at different temperature: (a) 5 mol%, (b) 10 mol%, (c) 15 mol%, (d) 20 mol%.

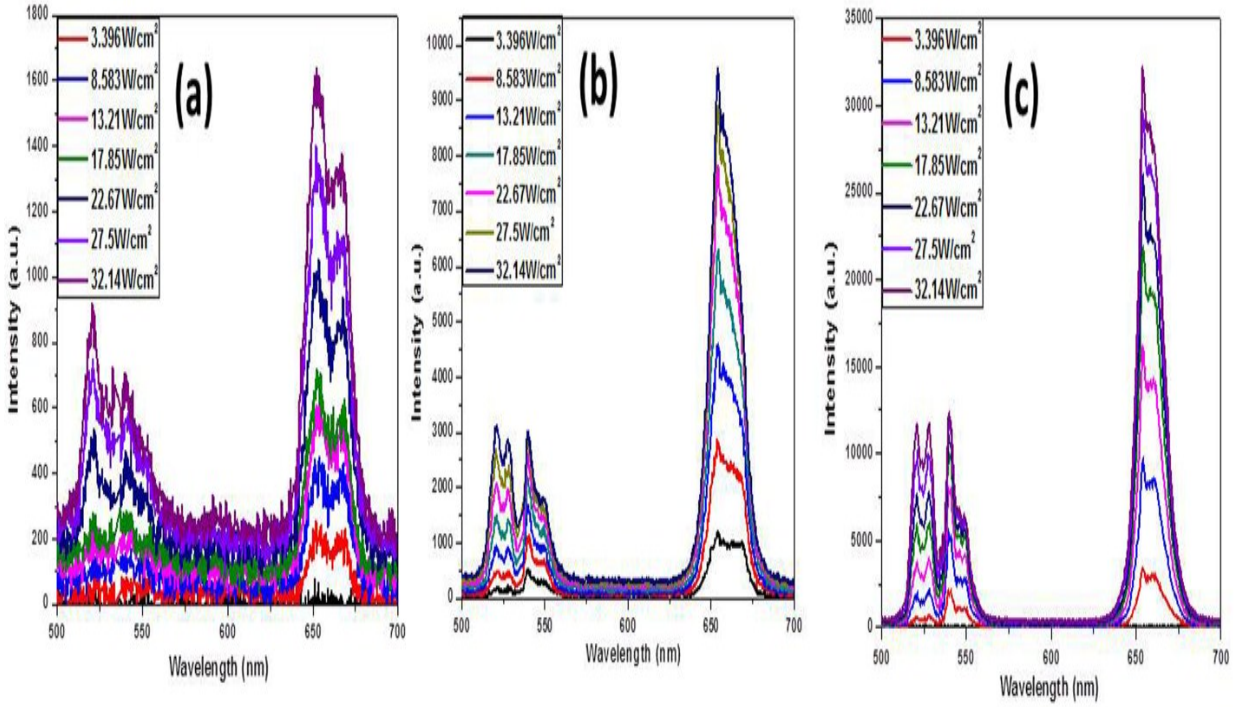

Fig.S5. Laser power dependence emission spectra of $NaYF_4$:Yb^{3+}/Er^{3+} crystals annealed at different temperature: (a) 200°C, (b) 400°C, (C) 600°C.

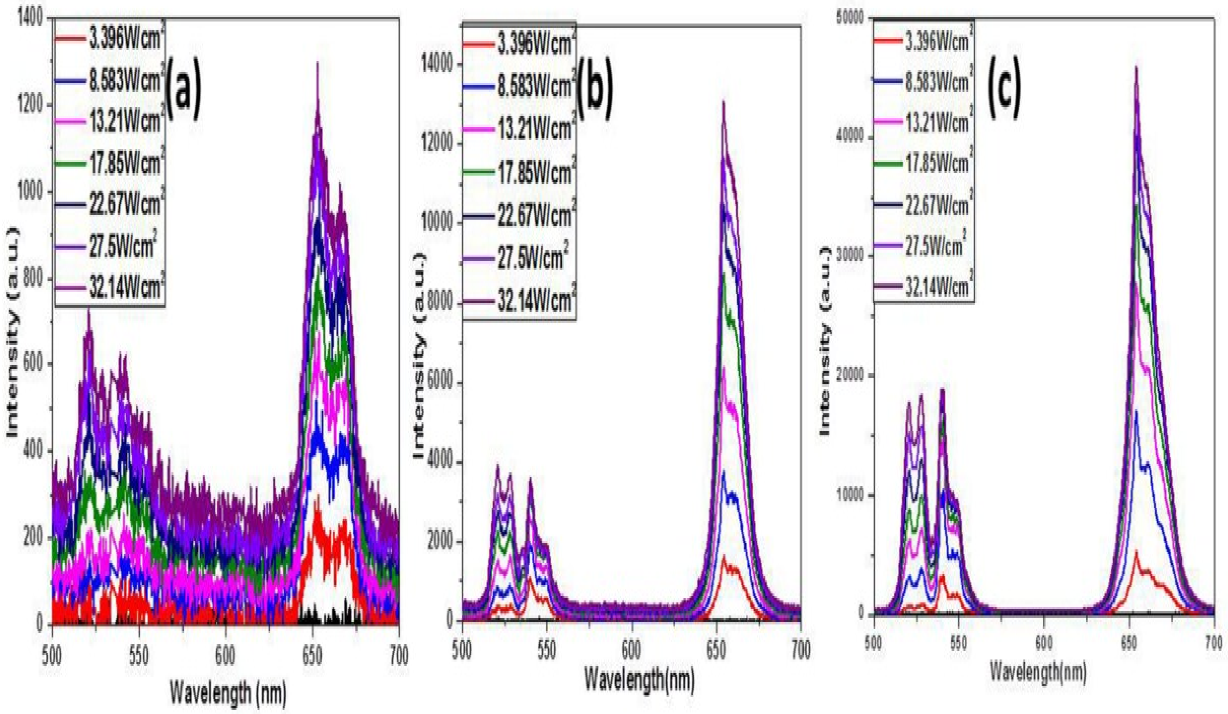

Fig.S6. Laser power dependence emission spectra of 5 mol% Cr^{3+} doped $NaYF_4$:Yb^{3+}/Er^{3+} microcrystals annealed at different temperature: (a) 200°C, (b) 400°C, (C) 600°C.

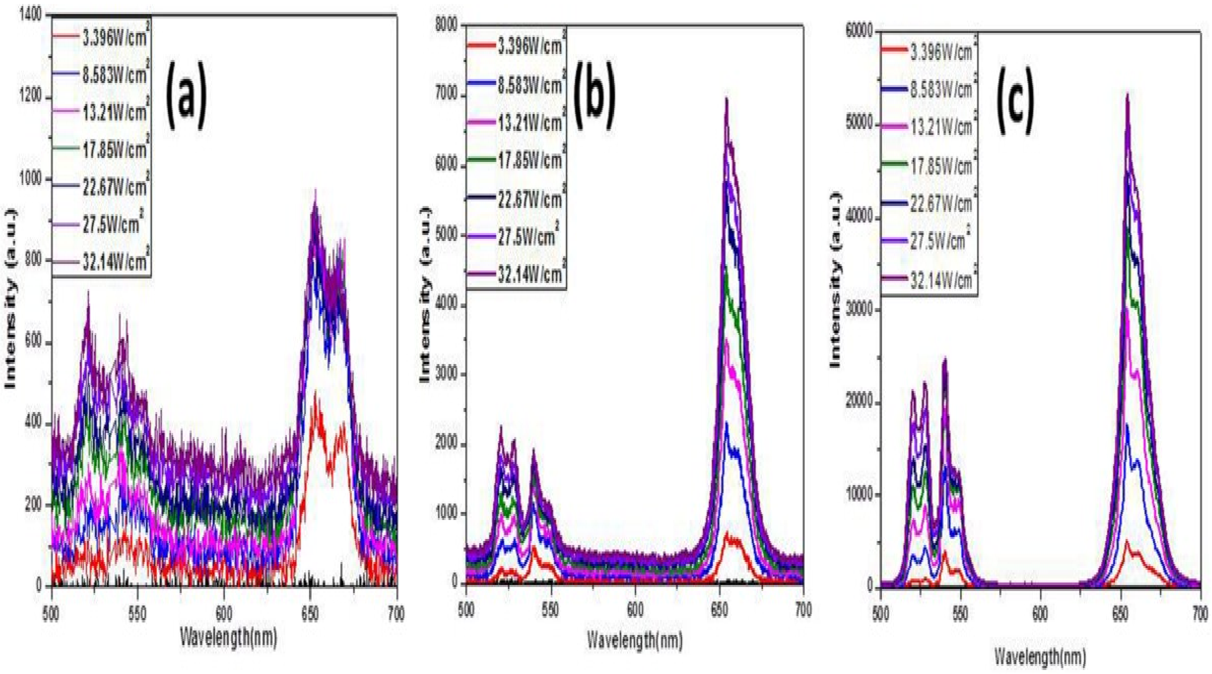

Fig.S7. Laser power dependence emission spectra of 10 mol% Cr^{3+} doped $NaYF_4$:Yb^{3+}/Er^{3+} crystals annealed at different temperature: (a) 200°C, (b) 400°C, (C) 600°C.

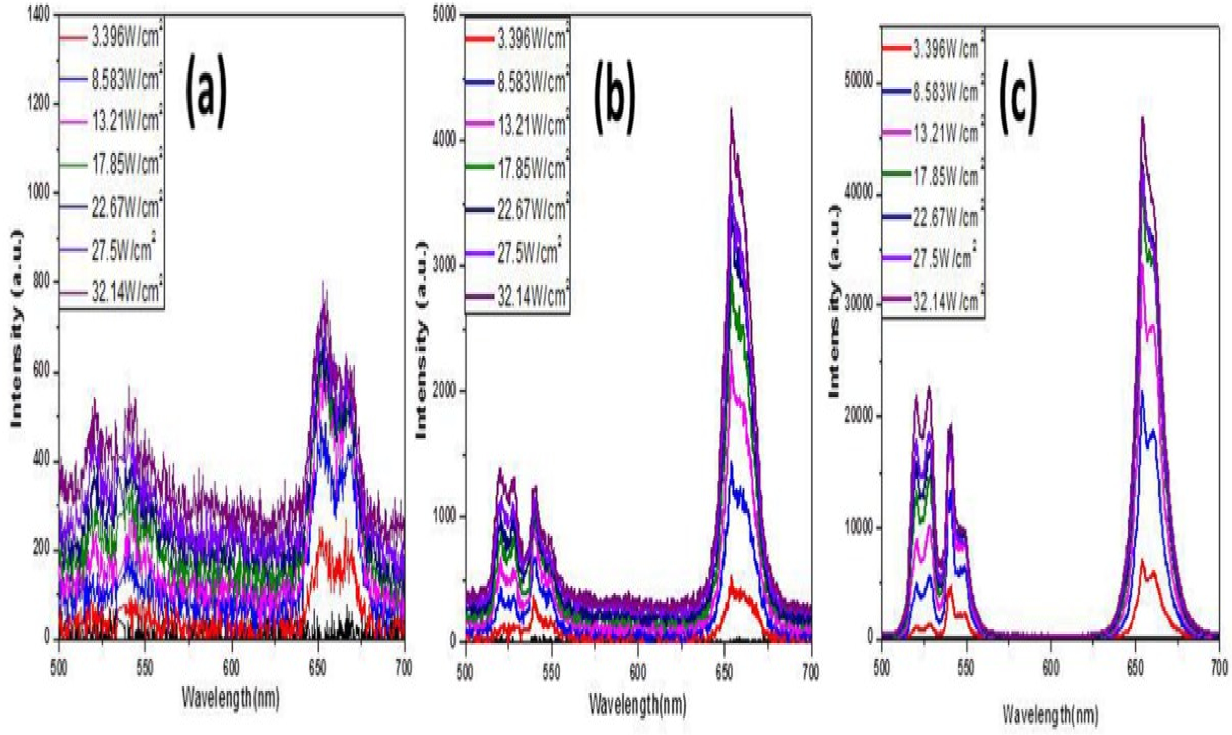

Fig.S8. Laser power dependence emission spectra of 15 mol% Cr^{3+} doped $NaYF_4$:Yb^{3+}/Er^{3+} crystals annealed at different temperature: (a) 200°C, (b) 400°C, (C) 600°C

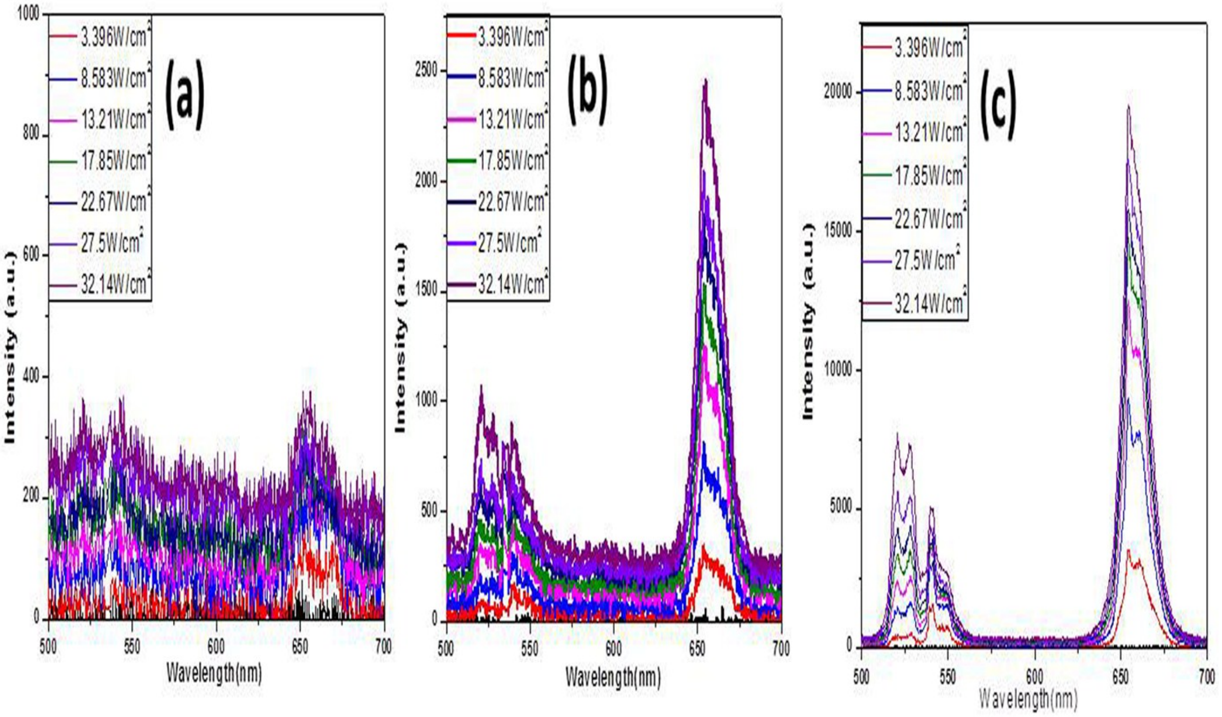

Fig.S9. Laser power dependence emission spectra of 20 mol% Cr^{3+} doped $NaYF_4$:Yb^{3+}/Er^{3+} crystals annealed at different temperature: (a) 200°C, (b) 400°C, (C) 600°C.
